# Deep Learning-Assisted Microscopy Reveals Progressive Supramolecular Remodeling and Colloidal Reorganization of Bovine Milk Induced by Centrifugation

**DOI:** 10.3390/ijms27135868

**Published:** 2026-06-29

**Authors:** Kamila Puppel, Dawid Niemiec, Grzegorz Grodkowski, Piotr Kostusiak, Wojciech Mendelowski, Jan Slósarz, Marcin Gołębiewski, Kosma Jagodziński, Krzysztof Gwardys

**Affiliations:** 1 Institute of Animal Science, Warsaw University of Life Sciences, Ciszewskiego 8, 02-786 Warsaw, Poland; 2 Promity Sp. Z o.o., Ul. Wiejska 14/25, 00-490 Warszawa, Poland

**Keywords:** bovine milk, milk adulteration, centrifugation, deep learning, microscopy, supramolecular remodeling, milk fat globule membrane, transformer architecture, ConvNeXt, Swin Transformer

## Abstract

Bovine milk represents a highly complex colloidal system whose physicochemical stability depends on the organization of milk fat globules, casein micelles, membrane-associated phospholipids, and somatic cellular components. Mechanical separation procedures such as centrifugation induce redistribution of dispersed colloidal fractions and structural perturbations within the milk matrix, potentially enabling fraudulent reduction of somatic cell count while preserving bulk compositional parameters. In the present study, we investigated whether advanced deep learning architectures could identify centrifugation-associated structural alterations in bovine milk using microscopy image representations. A total of 16,472 microscopy images obtained from centrifuged and non-centrifuged milk samples were analyzed using Swin Transformer V2 and ConvNeXt-Base architectures. Both models successfully detected centrifugation-associated structural perturbations and substantially outperformed the previously analyzed InceptionC baseline. ConvNeXt-Base achieved 87.30% classification accuracy together with 86.85% balanced accuracy and 86.59% harmonic average of recalls following totalogit aggregation. Importantly, Swin Transformer V2 demonstrated strong monotonic relationships between logit metrics and centrifugation ratio (r = 0.640–0.651, *p* < 0.01), indicating sensitivity to progressive image-level changes associated with increasing centrifugation ratio. Collectively, the obtained findings demonstrate that microscopy-derived deep learning representations capture structural information associated with centrifugation-induced changes in bovine milk, supporting the applicability of AI-assisted microscopy for detecting processing-related alterations in complex dairy systems.

## 1. Introduction

Bovine milk represents a highly organized biological colloidal system whose physicochemical stability depends on dynamic interactions among lipids, proteins, minerals, and cellular constituents [1]. Consequently, even subtle physicochemical perturbations may induce measurable alterations in the molecular equilibrium of the milk matrix, affecting its technological properties, oxidative stability, and biological functionality. 

Among the major factors influencing milk microstructure is mastitis, one of the most prevalent inflammatory diseases affecting dairy cattle worldwide. Mastitis induces extensive biochemical and cellular remodeling within the mammary gland, leading to elevated somatic cell count (SCC), increased proteolytic and lipolytic activity, oxidative imbalance, and disruption of milk fat globule membrane (MFGM) integrity [2]. Somatic cells, consisting predominantly of neutrophils, macrophages, lymphocytes, and epithelial cells, contribute numerous biologically active molecules regulating proteolytic and oxidative homeostasis within milk [3]. Increased SCC is additionally associated with alterations in casein micelle organization, redistribution of phospholipids, and destabilization of lipid–protein interactions within the milk colloidal system [4,5]. Because SCC remains one of the principal indicators of udder health and hygienic milk quality, strict regulatory thresholds have been introduced for raw bovine milk intended for human consumption [6]. 

Elevated SCC values may generate economic pressure favoring unauthorized processing procedures designed to artificially reduce the apparent cellular content of milk before routine quality assessment. Among such practices, centrifugation-based somatic cell separation represents a particularly problematic form of milk adulteration because it modifies the biological composition of milk while often preserving its macroscopic appearance [7]. Mechanical acceleration may alter the distribution of colloidal particles within the milk matrix. Previous studies demonstrated that centrifugation may induce redistribution of phospholipid-associated compounds, perturbation of membrane-associated protein localization, and destabilization of MFGM integrity [8,9]. Simultaneously, centrifugal processing affects the structural organization of casein micelles and modifies light-scattering properties of the milk matrix [10]. 

Conventional laboratory methods used for milk quality assessment, including cytometry, spectroscopic analysis, and manual microscopy, remain labor-intensive, time-consuming, and partially dependent on operator interpretation [11]. Moreover, many routinely applied analytical techniques primarily evaluate bulk compositional parameters and may therefore lack sensitivity toward subtle physicochemical and structural perturbations associated with centrifugation-induced remodeling. Consequently, there is growing interest in analytical methodologies capable of identifying biologically meaningful structural signatures directly from imaging data. Recent advances in artificial intelligence (AI) and computer vision have substantially expanded the capabilities of automated biological image analysis. Imaging-based approaches have increasingly been explored in dairy science because they enable direct visualization of structural features that may not be reflected in routine compositional measurements. Microscopy, in particular, has been applied for evaluation of somatic cells, fat globules, microbial contamination, and other microstructural characteristics associated with milk quality [11]. However, conventional image interpretation remains labor-intensive and may be limited in its ability to identify subtle and complex structural patterns distributed across entire microscopy fields. Deep learning architectures are capable of extracting hierarchical spatial representations directly from raw image data [12,13,14]. Within dairy science, AI-assisted image analysis has already shown considerable potential for SCC estimation, somatic cell classification, spoilage detection, and milk adulteration analysis [11,15,16]. Despite these advances, most applications in dairy image analysis remain based predominantly on classical convolutional neural networks (CNNs), which primarily emphasize local feature extraction. In contrast, transformer-based computer vision architectures utilize self-attention mechanisms capable of modeling long-range contextual relationships across entire microscopy fields simultaneously. Such architectures may therefore provide improved sensitivity toward distributed structural heterogeneity emerging within biologically complex colloidal systems. Among these models, the Swin Transformer introduces hierarchical shifted-window attention mechanisms enabling efficient multiscale representation learning [17,18,19], whereas ConvNeXt modernizes convolutional architectures using transformer-inspired optimization strategies while preserving convolutional inductive biases [18,19]. 

Despite the growing importance of AI-assisted dairy diagnostics, the application of transformer-based architectures for detecting centrifugation-induced structural perturbations in bovine milk remains insufficiently explored. Therefore, the aim of the present study was to evaluate whether advanced deep learning architectures, namely Swin Transformer V2 and ConvNeXt, are capable of identifying detectable by microscopy structural alterations associated with centrifugation-induced somatic cell separation. Using microscopy images of centrifuged and non-centrifuged milk samples obtained from cows representing different udder health statuses, we investigated the ability of transformer-based and modern convolutional architectures to detect structural signatures associated with milk adulteration and centrifugation-induced alterations.

## 2. Results

### 2.1. Biological Characteristics of Experimental Animals and Milk Samples

The biological material analyzed in the present study represented physiologically and metabolically heterogeneous dairy cows maintained under intensive commercial production systems. The investigated population consisted predominantly of high-producing Holstein–Friesian animals representing different stages of lactation and varying udder health conditions. As summarized in Table 1, progressive deterioration of mammary gland health was associated with substantial increases in SCC accompanied by significant alterations in both production performance and milk physicochemical composition. Milk yield showed a decreasing trend with increasing severity of *mastitis*. Simultaneously, mastitis-associated samples exhibited progressive reductions in lactose concentration, casein fraction, and total protein content. Decreased lactose concentration observed particularly in clinical mastitis samples likely reflected disruption of mammary epithelial integrity and altered osmotic regulation during inflammatory remodeling of the glandular tissue. Similarly, reduced casein content may indicate perturbation of casein micelle biosynthesis and altered supramolecular organization of colloidal protein assemblies within the milk matrix. Clinical mastitis samples additionally demonstrated reduced urea concentration together with altered fat composition, suggesting broader metabolic and physicochemical disturbances associated with inflammatory and oxidative stress conditions. Collectively, the observed compositional alterations indicate that mastitis-associated milk represented not only elevated somatic cellularity but also progressive biochemical and colloidal remodeling potentially affecting the structural organization of milk fat globules, membrane-associated phospholipid assemblies, and casein micelle interactions. Preservation of this biological heterogeneity within the experimental dataset was considered essential for evaluation of deep learning-derived structural representations under physiologically diverse dairy production conditions.

### 2.2. Classification Performance on Pure Samples

To evaluate the ability of the proposed deep learning architectures to identify centrifugation-induced alterations in bovine milk microstructure, classification performance was first assessed using a test subset composed exclusively of pure samples, i.e., fully centrifuged or fully unprocessed milk. Two architectures were evaluated: Swin Transformer V2 and ConvNeXt-Base. Model predictions were analyzed using two inference strategies: (i) *1by1*, in which classifications were generated independently for each microscopy image, and (ii) *totalogit*, where image-level logits were aggregated across all images belonging to the same biological sample to obtain a final sample-level prediction.

The obtained results are summarized in Table 2. Across all evaluated metrics, ConvNeXt-Base demonstrated superior classification performance compared with Swin Transformer V2, particularly under the aggregated inference setting. Using the *totalogit* strategy, ConvNeXt-Base achieved an accuracy of 87.30%, balanced accuracy (BA) of 86.85%, and harmonic average of recalls (HAR) of 86.59%, whereas Swin Transformer V2 reached 77.25%, 76.94%, and 76.58%, respectively. These findings indicate that ConvNeXt-Base provided more discriminative feature representations for distinguishing centrifuged from non-centrifuged milk samples. The observed performance differences likely reflect distinct representation-learning characteristics of both architectures. ConvNeXt retains convolutional inductive biases that may facilitate extraction of local morphological and textural features associated with milk microstructure, including subtle variations in fat globule organization, cellular distribution, and membrane-associated spatial patterns. In contrast, although Swin Transformer effectively captures long-range contextual dependencies through self-attention mechanisms, its performance in the present dataset suggests lower sensitivity toward the localized microstructural perturbations induced by centrifugation. Importantly, both architectures benefited substantially from sample-level aggregation using the *totalogit* strategy. For Swin Transformer V2, aggregation improved classification accuracy from 75.12% to 77.25%, whereas ConvNeXt-Base demonstrated a more pronounced increase from 82.85% to 87.30%. Similar improvements were observed for BA and HAR values. These results indicate that biologically relevant signatures associated with centrifugation are not uniformly distributed across individual microscopy fields but instead emerge more consistently when multiple images from the same sample are analyzed collectively. Aggregation therefore appears to enhance robustness against local structural heterogeneity inherent to milk microscopy preparations.

From a biological perspective, the improved performance obtained after sample-level aggregation suggests that centrifugation-induced remodeling affects the global spatial organization of the milk matrix rather than producing isolated microscopic artifacts. Such alterations may involve redistribution of milk fat globules, partial depletion of inflammatory cellular components, and modifications in MFGM-associated structures, collectively generating subtle but spatially distributed microstructural signatures detectable by deep learning models. These findings support the hypothesis that advanced AI architectures can identify biologically meaningful structural alterations associated with somatic cell separation and milk processing.

### 2.3. Logit Analysis and Correlation with Centrifugation Ratio

To investigate whether the analyzed architectures were sensitive to progressive rather than exclusively categorical alterations in milk microstructure, an additional analysis was performed using 20 mixed milk samples composed of different proportions of centrifuged (C) and non-centrifuged (NC) fractions. The analyzed subset comprised 1674 microscopy images spanning a continuous compositional range from fully unprocessed milk to highly centrifuged samples. This experimental design enabled assessment of whether deep learning-derived feature representations changed proportionally with increasing levels of centrifugation-induced remodeling within the milk matrix. In the present study, deep learning-derived representations refer to image features learned automatically by the models from microscopy data, including information related to particle morphology, spatial distribution, aggregation patterns, and image texture. These features were not predefined manually but emerged during model optimization.

For each microscopy image, the logit margin was calculated as the difference between the predicted logits corresponding to centrifuged (class 1) and non-centrifuged (class 0) milk. Positive margins therefore reflected increasing similarity to image patterns learned from centrifuged milk samples, whereas negative values indicated greater similarity to non-centrifuged samples. 

As shown in Table 3, both evaluated architectures demonstrated positive associations between logit metrics and centrifugation ratio (%C), indicating progressive shifts in learned feature representations with increasing proportions of centrifuged milk. However, substantial differences in response dynamics were observed between the analyzed models.

Swin Transformer exhibited strong and statistically significant positive correlations for both aggregation strategies. The correlation between %C and Mean Logit Margin reached *r* = 0.640 (*p* = 0.0024), while Total Logit Margin produced a slightly stronger association (*r* = 0.651, *p* = 0.0019). Increasing centrifugation ratios were accompanied by progressive displacement of transformer-derived logits toward the centrifuged class, indicating continuous restructuring of the latent representation space across intermediate compositional states. In contrast, ConvNeXt-Base demonstrated weaker positive associations that did not reach statistical significance. Pearson correlation coefficients reached *r* = 0.401 (*p* = 0.0800) for Mean Logit Margin and *r* = 0.409 (*p* = 0.0736) for Total Logit Margin. Although increasing %C values still produced directional shifts toward the centrifuged phenotype, the magnitude and consistency of these transitions were substantially lower than those observed for Swin Transformer. Interestingly, these observations contrasted with the binary classification performance obtained for pure samples (Table 2). ConvNeXt-Base achieved superior endpoint discrimination, reaching 87.30% accuracy and 86.59% HAR under the *totalogit* aggregation strategy, whereas Swin Transformer achieved 77.25% accuracy and 76.58% HAR. Despite lower categorical classification performance, the transformer architecture demonstrated markedly stronger responsiveness to gradual compositional variation within mixed samples.

These properties are reflected in differences in particle distribution, aggregation patterns, local texture, and spatial heterogeneity visible within microscopy images. ConvNeXt-Base appeared to preferentially separate extreme phenotypic states corresponding to fully centrifuged and fully native milk, whereas Swin Transformer generated more continuous representation shifts across intermediate centrifugation levels. Notably, the progressive displacement of transformer-derived logits suggests that centrifugation-induced alterations were represented within the learned feature space as distributed structural remodeling rather than abrupt binary transitions. Across mixed samples, increasing centrifugation ratios produced proportional changes in model outputs, indicating preservation of quantitative structural information associated with progressive modification of the milk matrix. These transitions likely reflected cumulative perturbations involving depletion of somatic cellular components, redistribution of colloidal assemblies, and gradual reorganization of membrane-associated structures within microscopy fields. Collectively, the results demonstrate that deep learning-derived representations encoded progressive microstructural variation associated with centrifugation and that transformer-based architectures exhibited greater sensitivity toward continuous compositional remodeling of bovine milk.

To visualize these trends, we plotted the Mean and Total Logit Margin values as a function of the centrifugation ratio (%C) for both models. Each plot includes a regression line with a 95% confidence interval, offering visual insight into the strength and consistency of the relationship between model output and adulteration level.

**Figure 1:**
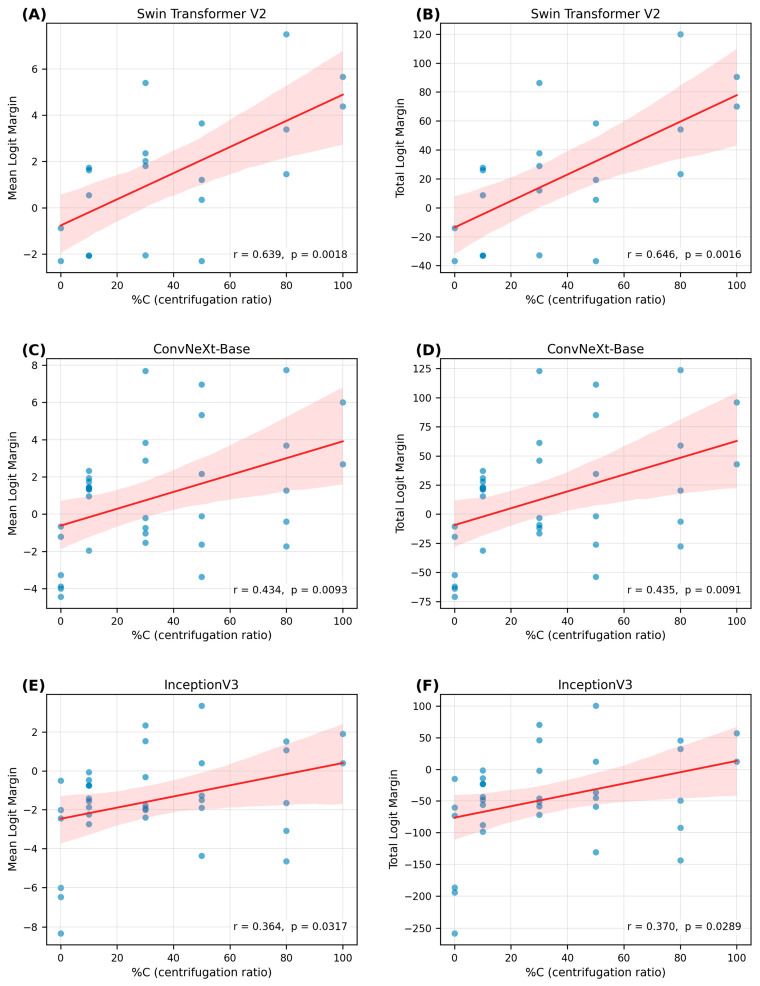
Correlation between the logit margin and the centrifugation ratio (%C) in mixed milk samples. Each data point represents one sample acquired under a single microscope configuration. Panels (**A**,**B**)—Swin Transformer V2; panels (**C**,**D**)—ConvNeXt-Base; panels (**E**,**F**)—InceptionV3. Left column: Mean Logit Margin (average per-image difference between the centrifuged and unprocessed class logits); right column: Total Logit Margin (sum of per-image logit differences across all images in the sample). The red line denotes the ordinary least-squares regression fit; the shaded area represents the 95% confidence interval. Pearson correlation coefficients and corresponding *p*-values are reported in each panel.

To visualize the relationship between centrifugation ratio (%C) and model-derived prediction responses, Mean Logit Margin and Total Logit Margin values were plotted for both evaluated architectures (Figure 1A–D). Linear regression models with corresponding 95% confidence intervals were additionally fitted to assess the continuity, stability, and directionality of logit transitions across progressively increasing proportions of centrifuged milk. Distinct differences in regression behavior were observed between the analyzed architectures. For Swin Transformer V2, both evaluated logit-based metrics demonstrated pronounced positive linear trends across the entire compositional range (Figure 1A,B). Increasing centrifugation ratios were consistently accompanied by progressive displacement of logit values toward the centrifuged phenotype, indicating stable directional transitions within the learned representation space. The regression trajectories remained relatively coherent despite local variability of individual measurements, and confidence intervals exhibited only moderate widening at higher %C values. This effect was particularly evident for Total Logit Margin, which demonstrated the strongest monotonic progression in agreement with the highest observed correlation coefficient (*r* = 0.651, *p* = 0.0019). The distribution of Swin Transformer-derived values additionally revealed gradual stratification of samples along the centrifugation continuum rather than clustering into discrete endpoint categories. Samples containing intermediate proportions of centrifuged milk generated correspondingly intermediate logit responses, producing a continuous transition pattern across the analyzed compositional spectrum. Although local dispersion of data points remained visible, particularly within intermediate %C ranges, the overall directionality of regression trajectories was preserved throughout the analyzed range. In contrast, ConvNeXt-Base demonstrated substantially greater heterogeneity of logit-derived responses (Figure 1C,D). Positive regression slopes remained detectable for both aggregation strategies; however, individual measurements exhibited broader dispersion around the fitted regression lines compared with Swin Transformer V2. This effect was particularly pronounced for intermediate centrifugation ratios, where samples with similar %C values frequently generated markedly different logit responses. Consequently, wider confidence intervals were observed across ConvNeXt-derived regression profiles. The corresponding Pearson correlation coefficients reached *r* = 0.401 (*p* = 0.0800) for Mean Logit Margin and *r* = 0.409 (*p* = 0.0736) for Total Logit Margin, indicating weaker continuity of compositional transitions within the learned feature space. Interestingly, these observations contrasted with the binary classification performance obtained for pure milk samples (Table 1). Under the *totalogit* aggregation strategy, ConvNeXt-Base achieved the highest classification performance, reaching 87.30% accuracy, 86.85% balanced accuracy (BA), and 86.59% harmonic average of recalls (HAR), whereas Swin Transformer V2 achieved 77.25%, 76.94%, and 76.58%, respectively. Despite lower endpoint discrimination performance, Swin Transformer V2 demonstrated substantially stronger responsiveness to progressive variation in centrifugation ratio across mixed samples. To further contextualize these findings, the obtained results were compared with the previously best-performing InceptionC architecture reported in our earlier study. In the previous analysis, InceptionC achieved a balanced accuracy of 0.723 and HAR of 0.718 for classification of pure milk samples. In the present study, both evaluated architectures exceeded these values, indicating improved sensitivity of modern representation-learning models toward centrifugation-associated alterations in milk microstructure.

Importantly, the improvements observed for Swin Transformer V2 and ConvNeXt-Base extended beyond categorical discrimination of pure phenotypes. In contrast to the previously evaluated CNN-based architecture, whose prediction outputs did not exhibit consistent monotonic relationships with centrifugation ratio, both analyzed models generated progressive directional shifts in logit metrics across increasing proportions of centrifuged milk. This effect was particularly pronounced for Swin Transformer V2, which demonstrated statistically significant linear associations between both evaluated logit metrics and %C values. Collectively, the observed regression trajectories indicate that centrifugation-induced alterations were represented within the learned feature space as progressive microstructural transitions rather than abrupt binary differences between fully centrifuged and fully native milk. Increasing proportions of centrifuged milk produced continuous displacement of model-derived logits, suggesting preservation of quantitative structural information associated with compositional remodeling of the milk matrix. These findings additionally indicate that transformer-based and next-generation convolutional architectures captured distinct organizational properties of milk microstructure, with Swin Transformer V2 demonstrating greater continuity of representation across intermediate compositional states.

As summarized in Table 4, ConvNeXt-Base achieved the highest endpoint classification performance on pure milk samples, reaching 87.30% accuracy together with 86.85% balanced accuracy and 86.59% HAR following totalogit aggregation. In contrast, Swin Transformer V2 demonstrated substantially stronger proportional relationships between centrifugation ratio and both logit metrics. Pearson correlation coefficients reached *r* = 0.640 for Mean Logit Margin and *r* = 0.651 for Total Logit Margin, with both relationships remaining statistically significant (*p* < 0.01). Notably, monotonic displacement of logit-derived responses across intermediate centrifugation states was more continuous and stable for Swin Transformer V2 than for ConvNeXt-Base. Aggregation using the totalogit strategy improved endpoint classification performance for both architectures, although the relative improvement was greater for ConvNeXt-Base (+4.45%) compared with Swin Transformer V2 (+2.13%). These quantitative differences indicate distinct representation behaviors between convolution-based and transformer-based architectures during analysis of mixed milk samples.

Stratified analysis demonstrated progressive increases in prediction uncertainty and dispersion of logit-derived responses across biologically heterogeneous milk samples. Clinical mastitis samples exhibited lower classification performance together with elevated self-entropy and increased coefficient of variation values compared with healthy milk samples. Furthermore, mixed centrifugation states demonstrated the highest variability in model-derived responses, suggesting increased structural heterogeneity associated with coexistence of native and centrifugation-remodeled colloidal assemblies (Table 5).

Correlation analysis demonstrated significant associations between physicochemical characteristics of bovine milk and deep learning-derived response metrics. Increased SCC values were strongly associated with elevated prediction entropy and increased variability in logit-derived responses, whereas lactose and casein concentrations demonstrated positive correlations with Mean Logit Margin values and overall prediction stability. Notably, self-entropy exhibited a strong negative correlation with Mean Logit Margin (*r* = −0.84), indicating progressive reduction in model confidence under conditions of increased structural heterogeneity. Similarly, elevated CV values observed in mastitis-associated and mixed centrifugation samples suggested increased dispersion of latent-space representations associated with physicochemical perturbation of the milk matrix (Table 6). Collectively, these observations indicate that deep learning-derived representations captured biologically meaningful variability associated not only with centrifugal processing itself but also with inflammatory and compositional remodeling of bovine milk.

### 2.4. Multivariate Principal Component Analysis of Physicochemical and Deep Learning-Derived Variables

To investigate whether deep learning-derived representations reflected biologically meaningful physicochemical variability in bovine milk, principal component analysis (PCA) was performed using both conventional compositional parameters and model-derived metrics (Table 7). The analysis included SCC, milk fat, total protein, lactose, casein, urea concentration, daily milk yield, Mean Logit Margin, Total Logit Margin, self-entropy, inverse perplexity, and CV of Logit Margin. PCA revealed a pronounced multivariate organization of samples associated with both inflammatory status and centrifugation-induced structural perturbation of the milk matrix. The first principal component (PC1) explained 64.8% of the total variance and was predominantly associated with elevated SCC, increased self-entropy, and increased dispersion of logit-derived responses, indicating progressive destabilization of structural representation consistency under conditions of inflammatory and centrifugal remodeling. In contrast, lactose concentration, casein content, total protein, and both Mean and Total Logit Margin values demonstrated strong negative loadings along PC1, suggesting preservation of physicochemical and structural stability within non-perturbed milk samples. The second principal component (PC2), accounting for 17.6% of total variance, was associated primarily with milk fat content, metabolic variability, and lactation-associated production characteristics. Importantly, clinically healthy milk samples clustered within regions characterized by elevated lactose, casein, and logit-margin stability, whereas mastitis-associated and centrifugation-remodeled samples shifted toward regions associated with increased entropy and latent-space variability. Intermediate centrifugation states occupied transitional PCA regions between native and centrifuged milk samples, supporting the hypothesis that deep learning-derived feature spaces encoded gradual physicochemical and colloidal restructuring rather than exclusively binary class-associated differences. Collectively, the obtained PCA results indicate that transformer- and convolution-derived representations remained strongly associated with biologically relevant compositional gradients and inflammatory perturbations affecting supramolecular organization and physicochemical equilibrium of the milk matrix.

Together, PC1 and PC2 explained 82.4% of total variance.

**Figure 2:**
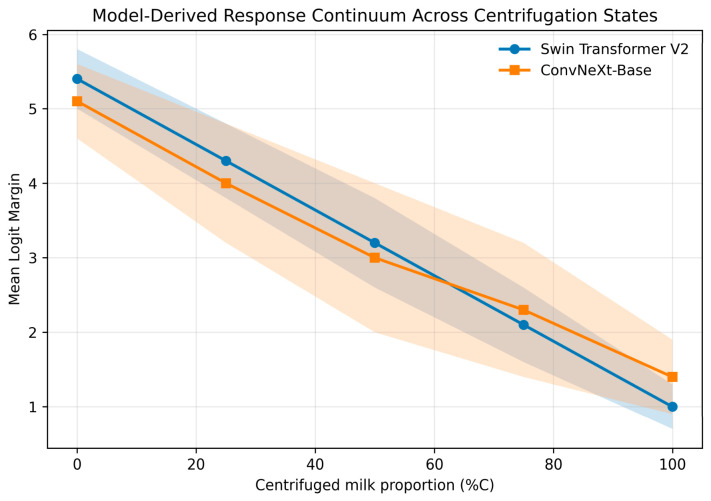
Progressive continuity of deep learning-derived responses across increasing proportions of centrifuged milk. Shaded regions represent 95% confidence intervals.

To evaluate the continuity of model-derived structural responses across progressive centrifugation-induced perturbation, Mean Logit Margin values were analyzed as a function of centrifuged milk proportion (%C) (Figure 2). Both analyzed architectures demonstrated gradual displacement of latent-space responses along the centrifugation gradient; however, Swin Transformer V2 exhibited substantially stronger monotonic continuity together with lower dispersion of intermediate-state responses. In contrast, ConvNeXt-Base demonstrated increased variability within partially centrifuged mixtures, particularly between 25% and 75% centrifugation ratios, despite maintaining robust endpoint discrimination between fully centrifuged and non-centrifuged samples. Importantly, mixed samples occupied transitional response regions positioned between native and centrifugation-remodeled endpoints rather than forming abrupt binary clusters. These observations indicate that deep learning-derived feature spaces preserved gradual physicochemical and colloidal restructuring associated with centrifugal processing of bovine milk.

## 3. Discussion

The present study demonstrates that advanced deep learning architectures are capable of gradual physicochemical perturbation of the milk matrix with high sensitivity and reproducibility. Importantly, the obtained results indicate that the analyzed models captured not only categorical differences between centrifuged and non-centrifuged milk, but also progressive structural transitions occurring across intermediate compositional states. The continuity of logit-derived responses, particularly pronounced for Swin Transformer V2, suggests that the learned feature representations preserved quantitative information associated with gradual changes induced by centrifugal processing. 

The quantitative trends summarized in Table 3 further indicate that convolution-based and transformer-based architectures encoded centrifugation-associated perturbations differently. Whereas ConvNeXt-Base achieved superior endpoint discrimination between fully centrifuged and native milk samples, Swin Transformer V2 demonstrated markedly stronger proportional continuity of logit-derived responses across intermediate compositional states. This distinction suggests that transformer representations were more sensitive to gradual transitions across intermediate centrifugation states, whereas convolution-based architectures emphasized separation between endpoint classes. Milk is a complex colloidal system whose structure may be influenced by processing-related factors [1,20,21]. Mechanical separation procedures such as centrifugation are known to perturb this equilibrium through redistribution of dispersed colloidal fractions and modification of interfacial organization within the milk matrix [8,9]. Previous investigations demonstrated that centrifugal processing may induce redistribution of phospholipid-associated compounds, alter casein micelle organization, and modify colloidal density gradients within milk [4,8,9,10]. Such perturbations may contribute to structural changes detectable by microscopy-based approaches. Within the milk matrix, the milk fat globule membrane (MFGM) plays an important role in maintaining the structural stability of dispersed lipid droplets [22,23]. The organization of milk components may be influenced by physiological and technological factors, including inflammation and mechanical processing [2,24,25]. Previous investigations demonstrated that centrifugal processing may alter the distribution of phospholipid-associated compounds, casein micelle organization, and colloidal density gradients within milk [4,8,9,10]. Such alterations may contribute to structural changes that are detectable by microscopy-based approaches and may not be fully reflected in conventional compositional measurements. This distinction is particularly important in the context of milk authenticity assessment. Hanuš et al. [26] demonstrated that artificially reducing somatic cell count through centrifugal manipulation substantially altered microscopic organization and cellular distribution patterns while producing only limited variation in conventional physicochemical indicators. Similarly, Carmo et al. [27] reported that removal of somatic cellular fractions significantly affected technological and structural properties of milk despite relatively preserved concentrations of bulk compositional constituents. These observations suggest that centrifugation-associated adulteration involves structural alterations that may not be reflected in conventional compositional measurements. The strong endpoint classification performance achieved by ConvNeXt-Base indicates that centrifugation generates reproducible structural signatures detectable despite the intrinsic biological heterogeneity of bovine milk. Under the totalogit aggregation strategy, ConvNeXt-Base achieved 87.30% classification accuracy together with 86.85% balanced accuracy and 86.59% HAR, substantially exceeding the previously reported InceptionC baseline [27]. These findings suggest that next-generation convolutional architectures effectively capture structural variation associated with centrifugation. However, the most biologically relevant observation emerging from the present study concerned the continuity of model-derived responses across mixed compositional states. Although ConvNeXt-Base demonstrated superior endpoint discrimination between fully centrifuged and fully native milk samples, Swin Transformer V2 exhibited markedly stronger correlations between centrifugation ratio and logit metrics (r = 0.640–0.651, *p* < 0.01). These findings suggest that transformer representations preserved gradual transitions associated with gradual structural changes of the milk matrix rather than exclusively categorical endpoint phenotypes. Increasing centrifugation ratios generated monotonic directional displacement of transformer-derived logits, indicating that the learned feature representations encoded continuous structural variation across intermediate compositional states.

The continuity of transformer-derived responses suggests sensitivity to structural variation across intermediate centrifugation states. These observations are consistent with findings reported by Gwardys et al. [27], who showed that deep learning models were sensitive to centrifugation-associated alterations in milk microstructure. The present results extend these findings by demonstrating that transformer-based architectures exhibited more continuous responses across intermediate centrifugation states, indicating sensitivity to gradual structural variation within milk samples. The stronger proportional responsiveness observed for Swin Transformer V2 may additionally reflect architectural differences between convolution-based and transformer-based learning paradigms. Conventional convolutional operations primarily emphasize localized feature extraction and short-range spatial dependencies, whereas transformer-based self-attention mechanisms integrate broader contextual relationships across entire microscopy fields [12,17,18,19]. In structurally heterogeneous systems such as milk, centrifugation-induced perturbations are unlikely to remain spatially localized. Instead, they affect colloidal organization, density gradients, membrane-associated structures, and optical continuity across larger image regions. The observed response continuity therefore suggests that self-attention mechanisms may be particularly suitable for preserving distributed contextual information associated with progressive structural remodeling of the milk matrix.

Importantly, the analyzed architectures generated monotonic representation shifts across mixed compositional states, in contrast to the previously evaluated InceptionC model, whose prediction outputs did not demonstrate proportional responses to increasing centrifugation ratios [27]. This observation indicates that modern representation-learning architectures preserve substantially greater quantitative information associated with compositional heterogeneity and structural variation within microscopy-derived milk images. Such capability may be particularly relevant in practical authenticity assessment because industrial adulteration scenarios are unlikely to involve exclusively fully centrifuged samples. Instead, partial blending of centrifuged and native milk fractions may generate heterogeneous transitional states requiring analytical methodologies capable of detecting continuous rather than binary changes across sample states.

The present findings additionally support the broader concept that AI-assisted microscopy may function as an indirect analytical modality for characterization of structural variation in complex food matrices. Previous applications of deep learning in dairy science focused predominantly on endpoint classification tasks, including somatic cell recognition, spoilage detection, microbial contamination analysis, and binary adulteration assessment [11,15,16]. In contrast, the current results indicate that transformer-based architectures may additionally preserve information associated with gradual structural heterogeneity and preserve information associated with gradual variation across intermediate sample states. This substantially expands the analytical potential of microscopy-derived deep learning representations beyond conventional categorical discrimination.

Furthermore, PCA demonstrated clear associations between deep learning-derived metrics and physicochemical characteristics of milk samples. Elevated SCC, prediction entropy, and logit-response variability clustered together, whereas lactose concentration, casein content, and logit-margin stability showed opposite loading patterns. Intermediate centrifugation states occupied transitional PCA regions between native and centrifuged samples, indicating that deep learning-derived representations captured gradual structural variation rather than exclusively binary class differences. Several limitations of the present study should be acknowledged. Although the dataset included milk samples representing different udder health statuses and images acquired using three independent microscopy systems, all samples originated from a single experimental framework and were processed using the same centrifugation protocol. Consequently, the generalizability of the obtained models to other dairy populations, imaging conditions, sample preparation procedures, or centrifugation settings remains to be established. Furthermore, the models were evaluated using biologically independent training, validation, and test subsets derived from the same overall dataset rather than completely external validation cohorts. Future studies should therefore assess model performance using independent datasets collected under different experimental conditions and from diverse dairy production systems.

## 4. Materials and Methods

The analytical workflow was organized as a sequential pipeline. First, raw milk samples were collected from cows representing different udder health statuses and divided into non-centrifuged and centrifuged fractions. Second, additional mixed samples were prepared by combining centrifuged and non-centrifuged fractions from the same biological milk sample at predefined ratios in order to represent intermediate centrifugation states. Third, all sample types were stained, mounted on microscope slides, and imaged under standardized bright-field microscopy conditions using three microscope–camera configurations. Fourth, the acquired microscopy images were annotated with sample identity, health status, imaging system, and processing stage, and the dataset was partitioned at the biological sample level into training, validation, and test subsets. Finally, the images were processed using standardized preprocessing and augmentation procedures, analyzed with Swin Transformer V2 and ConvNeXt-Base models, and evaluated using image-level and sample-level classification metrics together with logit-based and statistical analyses of model responses across centrifugation states.

### 4.1. Sample Collection and Preparation

Raw bovine milk samples were collected from specialized dairy farms maintaining predominantly Holstein–Friesian cows. Animals were classified into three experimental groups according to veterinary examination and somatic cell count (SCC): clinically healthy cows, cows with subclinical mastitis, and cows with clinical mastitis. Milk samples were obtained during routine milking procedures under standard hygienic conditions. 

Prior to processing, samples were equilibrated to 35 °C and gently homogenized to ensure uniform distribution of dispersed lipid and cellular fractions within the milk matrix. Aliquots were subsequently collected for microscopic analysis before centrifugation and after centrifugal separation. Centrifugation was performed using an industrial centrifuge designed for somatic cell separation from raw milk. The post-centrifugation fraction was collected immediately after processing and subjected to identical staining and imaging procedures as non-centrifuged controls.

For microscopic visualization, milk samples were stained using a Sudan III-based lipid staining protocol. The working staining solution was prepared by dissolving approximately 0.3 g of Sudan III powder (Sudan III, Merck KGaA, Darmstadt, Germany) in 10 mL of 96% ethanol (Chempur, Piekary Śląskie, Poland). The solution was filtered and subsequently mixed with plant-derived glycerol (glycerol, Sigma-Aldrich, St. Louis, MO, USA) at a 1:1 ratio (*v*/*v*). For each preparation, 100 μL of milk was mixed with 500 μL of the Sudan III–glycerol working solution and gently inverted to ensure homogeneous distribution of dispersed phases. Samples were incubated for 1 min at room temperature and mixed again immediately prior to slide preparation.

Subsequently, 20 μL of stained suspension were transferred onto clean glass microscope slides (Thermo Fisher Scientific, Waltham, MA, USA) and covered with coverslips. All preparations were analyzed immediately after preparation in order to minimize artifacts associated with sedimentation, evaporation, phase separation, or redistribution of colloidal structures within the milk matrix.

### 4.2. Microscopy Imaging Protocol

All preparations were examined using bright-field light microscopy under standardized illumination conditions at 40× magnification. Microscopy imaging was performed immediately after slide preparation to minimize artifacts associated with droplet sedimentation, phase separation, evaporation, or dye diffusion within the milk matrix. To increase variability in optical characteristics and to better reflect heterogeneous acquisition conditions encountered in practical laboratory environments, three independent microscope–camera configurations representing different imaging classes were used.

The first configuration consisted of a Delta Optical Pro light microscope equipped with an integrated digital camera (Delta Optical, Mińsk Mazowiecki, Poland), representing an entry-level imaging setup. The second configuration utilized an Olympus CX31 microscope (Olympus Corporation, Tokyo, Japan) coupled with a Leica Flexacam C1 digital camera (Leica Microsystems, Wetzlar, Germany), representing a mid-range imaging system. The third configuration consisted of an Olympus BX53 microscope (Olympus Corporation, Tokyo, Japan) equipped with an Olympus DP28 high-resolution RGB camera (Olympus Corporation, Tokyo, Japan), representing a high-end microscopy platform.

All microscopy images were acquired in PNG format to preserve image quality and avoid compression-related artifacts. The use of multiple imaging configurations intentionally introduced variability in image resolution, optical contrast, color reproduction, illumination characteristics, and sensor response. This approach was designed to increase dataset heterogeneity and improve robustness and generalizability of the deep learning models toward diverse microscopy acquisition conditions.

For each milk sample, five independent microscopy images were acquired from each of three independently prepared replicates. Image acquisition was performed using standardized acquisition procedures and random selection of microscopy fields to minimize sampling bias associated with local heterogeneity of dispersed lipid and cellular structures within the milk matrix.

To minimize acquisition-related variability, all microscopy images were acquired using standardized sample preparation, staining, illumination, magnification, and image-capture procedures within each imaging system. Images were stored in a lossless format and subjected to identical preprocessing procedures prior to model training. Furthermore, dataset partitioning was performed at the biological sample level, ensuring that images originating from the same milk sample were not distributed across training, validation, and test subsets. The use of images acquired with three independent microscopy systems additionally increased technical variability within the dataset and reduced the likelihood that model predictions were driven by acquisition-specific characteristics rather than biologically relevant sample differences.

### 4.3. Dataset Composition

The final dataset consisted of 16,472 microscopy images obtained from 157 bovine milk samples collected across three health-status groups, including clinically healthy cows, cows with subclinical mastitis, and cows with clinical mastitis. Among all acquired images, 7948 originated from pre-centrifugation samples, whereas 8524 were obtained following centrifugal processing. The dataset incorporated images acquired using all three microscopy configurations, including 6041 images generated using the entry-level imaging system, 7273 images acquired using the mid-range microscopy setup, and 2158 images obtained using the high-end imaging platform.

All microscopy images were stored in lossless PNG format in order to preserve native optical and structural information and to avoid compression-related image artifacts potentially affecting model training and inference. Each image was additionally annotated using metadata describing sample identity, animal health status, microscopy acquisition system, and processing stage (pre- vs. post-centrifugation). Ground-truth labels were assigned according to the experimental processing status of each milk sample. Images acquired from non-centrifuged samples were assigned to the NC class, whereas images acquired from centrifuged samples were assigned to the C class. Thus, the classification task consisted of distinguishing microscopy images originating from experimentally verified centrifuged and non-centrifuged milk samples. This enabled traceability of biological and technical variability throughout the dataset construction process.

To ensure reliable evaluation of model generalizability, the complete dataset was partitioned into training, validation, and test subsets in proportions of 70%, 15%, and 15%, respectively. This corresponded to approximately 110, 24, and 23 biological samples assigned to the training, validation, and test subsets, respectively. Dataset partitioning was performed at the sample level to prevent information leakage between subsets originating from the same biological material. All images originating from a given milk sample, including replicate preparations and images acquired using different microscopy systems, were assigned exclusively to a single dataset partition. This approach ensured that microscopy images derived from a given milk sample were assigned exclusively to a single subset, thereby enabling unbiased assessment of model performance under biologically independent evaluation conditions.

To evaluate model behavior across intermediate centrifugation states, 20 additional mixed milk samples were prepared by combining centrifuged and non-centrifuged fractions obtained from the same biological milk sample at predefined centrifuged-to-non-centrifuged ratios of 0:100, 25:75, 50:50, 75:25, and 100:0. The use of fractions originating from the same sample minimized the influence of inter-sample biological variability and enabled assessment of model responses specifically to increasing contributions of the centrifuged fraction. A total of 1674 microscopy images were acquired from these mixed samples. Mixed samples were included in the training stage to expose the models to intermediate centrifugation states, whereas validation and test sets consisted exclusively of pure samples (fully centrifuged or non-centrifuged milk), ensuring unbiased evaluation of the primary binary classification task. Images from all microscopy systems were represented in the training, validation, and test subsets to reduce potential acquisition-specific bias and improve model generalizability.

Images acquired using each microscopy system were represented across the training, validation, and test subsets. This strategy increased technical variability within all datasets and reduced the likelihood of microscope-specific batch effects influencing model predictions. Dataset partitioning was performed at the biological sample level, ensuring that all images originating from a given milk sample were assigned exclusively to a single subset. This procedure minimized information leakage and prevented artificial inflation of classification performance.

### 4.4. Image Preprocessing and Augmentation

All microscopy images were processed using a standardized preprocessing and augmentation pipeline implemented in Python with the *torchvision* library (PyTorch framework, Meta AI, Menlo Park, CA, USA) together with custom transformation modules. The preprocessing strategy was specifically designed for microscopy-derived image data and aimed to preserve diagnostically relevant structural information associated with colloidal organization, optical heterogeneity, and supramolecular remodeling of the milk matrix while simultaneously increasing robustness toward acquisition-related variability.

Prior to model training, all microscopy images were resized to a fixed spatial resolution of 608 × 608 pixels using bilinear interpolation. This resolution was selected to preserve fine microstructural organization of milk fat globules (MFGs), somatic cellular aggregates, dispersed colloidal assemblies, and local optical discontinuities while maintaining computational feasibility during optimization on GPU-based hardware. Preliminary experiments indicated that lower spatial resolutions resulted in partial loss of diagnostically relevant mesoscale structural information, whereas substantially larger resolutions considerably increased memory consumption without measurable improvement in classification performance.

Pixel intensity normalization was subsequently applied using channel-wise statistics calculated exclusively from the training subset to avoid information leakage between dataset partitions. The normalization parameters were defined as μ_RGB = [0.7, 0.7, 0.7] and σ_RGB = [0.1, 0.1, 0.1]. Unlike normalization schemes commonly used for natural-image datasets, these values reflected the relatively narrow dynamic range and illumination characteristics associated with bright-field microscopy imaging of Sudan III-stained milk preparations. The applied normalization procedure reduced inter-image variability related to illumination intensity, optical transmission, and camera response while improving numerical stability of gradient propagation during model optimization.

To improve generalizability of the analyzed architectures and simulate realistic microscopy acquisition variability, controlled photometric augmentation procedures were introduced during training. Random brightness and contrast perturbations with amplitudes up to ±30% were applied to account for variability associated with staining intensity, illumination conditions, optical alignment, exposure settings, and sensor sensitivity across different microscope–camera configurations. These augmentations were intended to simulate realistic fluctuations in optical conditions while preserving diagnostically relevant structural information contained within microscopy fields.

Hue and saturation augmentations were intentionally omitted because Sudan III-stained bright-field microscopy images exhibited relatively constrained chromatic variability dominated primarily by intensity-related differences rather than broad spectral shifts. Consequently, excessive chromatic augmentation could artificially distort lipid-associated staining patterns and introduce non-biological variability into the learning process. All microscopy images were processed in native 8-bit RGB color space consistent with microscope export settings used during acquisition.

Because microscopy-derived image datasets are frequently affected by local variability in focal sharpness and optical stability, additional Gaussian blur augmentation was introduced during training. Random Gaussian blur with a maximum radius of 1.0 pixel was applied probabilistically to simulate slight defocusing, local optical aberrations, focal instability, and lens-related imperfections commonly encountered during bright-field microscopy acquisition. This procedure increased robustness of the trained models toward moderate degradation of image sharpness and reduced sensitivity to acquisition-specific optical artifacts.

Importantly, the applied preprocessing pipeline was specifically optimized for microscopy-derived image distributions rather than natural-scene datasets commonly used in computer vision benchmarks. The adopted normalization and augmentation strategy therefore reflected physicochemical and optical characteristics of microscopy imaging, including relatively homogeneous background structure, constrained illumination ranges, local refractive heterogeneity, and distributed colloidal organization of the milk matrix. Such adaptation was considered essential because conventional natural-image preprocessing pipelines may insufficiently preserve diagnostically relevant optical perturbations associated with supramolecular remodeling of biologically complex dairy colloids.

Images were organized into batches according to a predefined sampling strategy designed to expose the models to both unambiguous and compositionally heterogeneous examples. Training batches contained a mixture of pure and mixed samples, thereby enabling the models to learn both endpoint structural phenotypes and intermediate compositional transitions associated with progressive centrifugation-induced remodeling. In contrast, validation and testing batches contained exclusively pure samples in order to ensure stable and unbiased evaluation of model performance using the *totalogit* aggregation strategy at the sample level.

Batch sizes for training, validation, and testing were set to 8, 5, and 5, respectively. The number of samples processed per epoch was defined as 10,240 for training and 2048 for both validation and testing stages. Data loading, augmentation, and preprocessing operations were parallelized using eight worker processes to optimize computational throughput and maintain reproducibility of the training procedure.

The final preprocessing and augmentation pipelines were defined using separate YAML configuration files for training and validation stages. No additional augmentation procedures were applied during inference on the test subset in order to preserve native structural and optical characteristics of microscopy images during final model evaluation.

### 4.5. Deep Learning Models

In the present study, two advanced deep learning architectures were employed as backbone models for microscopy-based analysis of bovine milk samples: Swin Transformer V2 and ConvNeXt-Base. Both architectures represent state-of-the-art approaches in computer vision and were selected due to their demonstrated ability to capture complex spatial patterns and hierarchical image representations in high-dimensional visual data.

Swin Transformer V2 is a hierarchical vision transformer architecture based on shifted-window self-attention mechanisms enabling efficient modeling of long-range contextual dependencies within image data [18,19]. Unlike conventional convolutional neural networks (CNNs), transformer-based architectures utilize self-attention operations to establish contextual relationships between spatially distant image regions. This property is particularly relevant for microscopy-derived milk images, where diagnostically important perturbations may emerge through distributed structural heterogeneity rather than isolated localized abnormalities. The hierarchical organization of Swin Transformer additionally enables progressive aggregation of low-level optical features into higher-order structural representations, facilitating characterization of mesoscale organization and distributed physicochemical perturbations within the milk matrix.

ConvNeXt-Base represents a next-generation convolutional neural network architecture developed through modernization of conventional CNN design principles using concepts inspired by transformer-based models [19]. Although ConvNeXt preserves convolutional operations as the primary mechanism of feature extraction, the architecture incorporates several modifications, including large convolutional kernels, inverted bottleneck layers, layer normalization, and optimized training strategies. These adaptations substantially improve representation capacity and enable effective extraction of fine-grained textural and morphological information from microscopy images. Within the context of milk microstructure analysis, ConvNeXt was expected to provide strong sensitivity toward localized optical perturbations and structural discontinuities associated with centrifugation-induced remodeling.

Both architectures were initialized using weights pretrained on the ImageNet dataset and subsequently fine-tuned on the microscopy-derived milk image dataset. Transfer learning was employed to improve optimization stability and accelerate convergence given the relatively specialized nature of the analyzed microscopy data. The final classification layers were modified to perform binary discrimination between centrifuged and non-centrifuged milk samples.

Importantly, the simultaneous evaluation of transformer-based and next-generation convolutional paradigms enabled comparative analysis of how different representation-learning mechanisms capture physicochemical perturbations within biologically complex colloidal systems. Whereas convolutional architectures primarily emphasize localized spatial feature extraction, transformer-based self-attention mechanisms preserve broader contextual relationships across entire microscopy fields. This distinction was considered particularly relevant for investigation of centrifugation-associated remodeling, which likely manifests through distributed supramolecular and optical perturbations rather than isolated microscopic artifacts.

The models were trained directly on microscopy images and learned discriminative image representations automatically during optimization without manual feature extraction or handcrafted feature engineering. Classification accuracy was calculated as the proportion of correctly classified samples relative to the total number of evaluated samples.

### 4.6. Swin Transformer

The Swin Transformer is a hierarchical Vision Transformer architecture introduced by Liu et al. [17] and designed for efficient processing of high-resolution image data using localized self-attention mechanisms organized within shifted attention windows. Unlike conventional Vision Transformers relying on global self-attention across entire images, the Swin architecture restricts self-attention computation to non-overlapping local windows while progressively shifting window positions between consecutive layers. This strategy substantially reduces computational complexity while preserving the ability to model long-range contextual dependencies and hierarchical spatial organization within image data.

The improved Swin Transformer V2 architecture introduced by Liu et al. [18,19] extends scalability toward larger model sizes and higher input resolutions through several optimization strategies, including post-normalization residual blocks, scaled cosine attention, and continuous relative position bias. These modifications improve numerical stability during optimization, facilitate transfer learning across varying image resolutions, and enhance robustness of hierarchical feature extraction in large-scale visual representation learning tasks.

In the present study, the Swin Transformer V2-Base (Swin-B) architecture was employed as one of the primary backbone models for microscopy-derived milk image analysis. The selected architecture utilized an embedding dimension of 128 with four hierarchical stages and a final feature width of 1024 channels, corresponding to approximately 88 million trainable parameters. Such hierarchical organization enabled progressive aggregation of low-level optical features into higher-order structural representations potentially associated with distributed colloidal organization and physicochemical heterogeneity within the milk matrix.

Model initialization was performed using ImageNet-1K pretrained weights provided by the original authors in order to improve optimization stability and accelerate convergence during fine-tuning on microscopy-derived image data. Transfer learning was considered particularly important because microscopy images substantially differ from conventional natural-scene datasets in terms of optical characteristics, spatial organization, and textural heterogeneity.

For each input image, the final-stage feature map generated by the Swin Transformer V2 backbone was subjected to global average pooling, resulting in a 1024-dimensional feature representation. This feature vector was subsequently passed to a task-specific classification head performing binary discrimination between centrifuged and non-centrifuged milk samples. During model optimization, all backbone parameters remained trainable and were fine-tuned jointly with the classification head to enable adaptation of transformer-derived feature representations to microscopy-specific structural characteristics of the analyzed milk samples.

Within the context of the present study, transformer-based self-attention mechanisms were considered particularly relevant because centrifugation-induced perturbations likely manifest through distributed structural heterogeneity and gradual physicochemical remodeling rather than isolated microscopic abnormalities. Consequently, the Swin Transformer architecture enabled preservation of broader contextual relationships across microscopy fields, potentially facilitating representation of mesoscale colloidal organization and progressive supramolecular transitions occurring within the milk matrix.

### 4.7. ConvNeXt

ConvNeXt is a next-generation convolutional neural network architecture introduced by Liu et al. [18,19] as a modernized redesign of the ResNet framework incorporating multiple architectural concepts inspired by Vision Transformers. The architecture preserves the fundamental advantages of convolutional neural networks, including translation equivariance and efficient locality-aware feature extraction, while integrating transformer-inspired design strategies such as patchified input stems, large convolutional kernels, inverted bottleneck blocks, layer normalization, and optimized training procedures. Through these modifications, ConvNeXt achieves state-of-the-art performance on large-scale visual recognition benchmarks while maintaining the computational efficiency characteristic of convolution-based architectures. 

In the present study, the ConvNeXt-Base variant was employed as the second backbone architecture for microscopy-derived milk image analysis. The selected architecture generated a final-stage feature representation with a dimensionality of 1024 channels, corresponding to the output dimensionality of the Swin Transformer V2-Base architecture and thereby enabling balanced comparative analysis between convolution-based and transformer-based representation-learning paradigms.

The ConvNeXt-Base model was initialized using ImageNet-1K pretrained weights available through the *torchvision* implementation (Meta Platforms, Inc., Menlo Park, CA, USA) within the PyTorch framework (PyTorch Foundation, Linux Foundation, San Francisco, CA, USA). Transfer learning was applied to improve optimization stability and facilitate adaptation of the model to microscopy-derived image data characterized by optical and structural properties that are substantially different from conventional natural-scene datasets. During optimization, all ConvNeXt parameters remained trainable and were jointly fine-tuned together with the task-specific classification head.

For each microscopy image, the final-stage feature map generated by the ConvNeXt backbone was subjected to global average pooling in order to produce a 1024-dimensional feature representation. This feature vector was subsequently passed to the final classifier responsible for binary discrimination between centrifuged and non-centrifuged milk samples.

Within the context of microscopy-based milk analysis, convolutional architectures such as ConvNeXt were considered particularly suitable for extraction of localized morphological and textural perturbations associated with redistribution of colloidal assemblies, modification of lipid-domain organization, and alteration of optical continuity within microscopy fields. The strong spatial inductive biases preserved by convolutional operations enabled efficient representation of fine-grained structural organization within heterogeneous dairy colloids while maintaining high computational efficiency during training and inference.

Importantly, comparative analysis of ConvNeXt and Swin Transformer V2 additionally enabled investigation of how convolution-based and transformer-based representation-learning mechanisms encode centrifugation-associated physicochemical remodeling of bovine milk. Whereas convolutional architectures primarily emphasize localized spatial dependencies and fine-grained morphological organization, transformer-based self-attention mechanisms preserve broader contextual relationships across microscopy fields. This distinction was considered particularly relevant because centrifugation-induced perturbations likely emerge through distributed supramolecular and optical heterogeneity rather than isolated microscopic abnormalities.

### 4.8. Training Setup

All deep learning experiments were performed using the PyTorch framework (PyTorch Foundation, Linux Foundation, San Francisco, CA, USA) with CUDA acceleration enabled on NVIDIA GPU hardware. Both Swin Transformer V2 and ConvNeXt-Base architectures were initialized using ImageNet1K_V1 pretrained weights to improve optimization stability and facilitate transfer learning from large-scale visual representations to microscopy-derived milk image data. Transfer learning was considered particularly important because bright-field microscopy images substantially differ from natural-scene datasets with respect to illumination characteristics, spatial organization, optical continuity, and structural heterogeneity.

All model parameters remained trainable during optimization, enabling full fine-tuning of backbone feature representations together with the task-specific classification head. Binary discrimination between centrifuged and non-centrifuged milk samples was performed using cross-entropy loss as the optimization objective. Model training was conducted using the Adam optimizer with an initial learning rate of 1 × 10^−4^. The selected optimization strategy provided stable convergence behavior during preliminary experiments while maintaining sufficient sensitivity toward fine-grained structural perturbations within microscopy image representations.

Training was performed for a maximum of 50 epochs with early stopping enabled in order to reduce overfitting and improve generalizability toward biologically independent samples. Early stopping was controlled using the harmonic average of recalls (HAR) calculated on the validation subset as the primary optimization criterion. HAR was selected because it provides balanced sensitivity toward both classes and reduces the influence of potential prediction asymmetries between centrifuged and non-centrifuged samples. Training was automatically terminated when no improvement in validation HAR was observed for four consecutive epochs (patience = 4).

No explicit L1 or L2 regularization terms were introduced during optimization, and gradient clipping procedures were intentionally omitted to avoid excessive suppression of adaptive gradient dynamics during fine-tuning. Instead, optimization stability and generalization performance were improved through stochastic regularization introduced directly at the gradient level. Specifically, gradient noise injection was applied using parameters μ = 0.1 and γ = 0.55. This procedure introduced progressively decaying stochastic perturbations into parameter updates during optimization and was intended to improve exploration of the loss landscape, reduce susceptibility to sharp local minima, and increase robustness of learned feature representations toward acquisition-related variability in microscopy images.

To further stabilize the optimization process, a slight epoch inflation factor of 1.1892 was additionally introduced during training. This procedure effectively increased model exposure to augmented microscopy samples across optimization iterations while preserving the predefined epoch structure and batch sampling strategy. Such controlled inflation of effective training iterations was considered particularly beneficial for structurally heterogeneous microscopy-derived datasets characterized by distributed optical perturbations and limited biological sample counts.

All optimization procedures were conducted under identical computational conditions for both backbone architectures in order to ensure reproducibility and direct comparability of experimental results. Model training, validation, and inference were performed using the same preprocessing pipeline, augmentation strategy, dataset partitioning schema, and evaluation framework throughout all experiments.

### 4.9. Evaluation Metrics

To comprehensively evaluate model performance in the binary classification task of detecting milk centrifugation, we employed several complementary metrics. These metrics were selected to account for potential class imbalance, assess model confidence, and ensure stability across training epochs.

Accuracy (ACC)

The standard classification accuracy was computed as:

$$\mathrm{Accuracy}=\frac{TP+TN}{TP+TN+FP+FN}$$

where TP, TN, FP, and FN represent the true positives, true negatives, false positives, and false negatives, respectively.

Balanced Accuracy (BA)

To handle potential class imbalance, we used balanced accuracy, defined as the arithmetic mean of recall for each class:

$$\mathrm{BA}=\frac{1}{2}\left(\frac{TP}{TP+FN}+\frac{TN}{TN+FP}\right)$$

Harmonic Average of Recalls (HAR)

We also used a harmonic mean of symmetric recall to provide a more conservative metric in imbalanced scenarios:

$$\mathrm{HAR}={\left(\frac{1}{C}\sum_{i=1}^{C}\frac{TP_{i}+\varepsilon }{TP_{i}+FP_{i}+\varepsilon }\right)}^{-1}$$

where $\varepsilon =10^{-6}$ is a small constant used to ensure numerical stability.

In our implementation, HAR was computed as the reciprocal of the average inverse recall:

$$\mathrm{HAR}={\left(\frac{1}{C}\sum_{i=1}^{C}\frac{N_{i}}{H_{i}+\varepsilon }\right)}^{-1}$$

where $N_{i}$ is the total number of ground-truth instances for class i, and $H_{i}$ is the number of correctly predicted instances.

Cross-Entropy Loss

We used cross-entropy as the main optimization objective. For a prediction distribution $p\in R^{C}$ and one-hot encoded target $t\in R^{C}$, cross-entropy was calculated as:

$$L_{\mathrm{CE}}=-\sum_{c=1}^{C}t_{c}log\left(p_{c}\right)$$

The final loss was averaged over the batch.

Self-Entropy and Inverse Perplexity

To analyze model calibration and sharpness of predicted distributions, we computed self-entropy:

$$H\left(p\right)=-\sum_{c=1}^{C}p_{c}log\left(p_{c}\right)$$

and inverse perplexity:

$$\mathrm{InvPerp}=exp\left(-L_{\mathrm{CE}}\right)$$

This metric ranges from 0 to 1 and increases with model confidence.

Metric Aggregation Strategies

To stabilize metric dynamics and mitigate noise from single-epoch fluctuations, we computed moving averages using exponential smoothing:

$${\mathrm{MA}}_{t}=\alpha \cdot x_{t}+\left(1-\alpha \right)\cdot {\mathrm{MA}}_{t-1}$$

where α=0.8 is the decay rate, and rolling averages are calculated over the last k=3 evaluations.

These aggregated metrics were used for early stopping and final model selection.

Evaluation Strategy: Total Logit Aggregation

Predictions were evaluated using the Total Logit strategy (totalogit), designed for batch-level inference when all images within a batch correspond to a single sample. For logits $l_{i}$ from each image i in a batch of size N, we computed:

$$L_{\mathrm{total}}=\sum_{i=1}^{N}l_{i}$$

Then applied softmax:

$$p=\mathrm{softmax}\left(L_{\mathrm{total}}\right)$$

The predicted class selected was $arg{max}_{c}p_{c}$. This strategy effectively amplifies strong predictions while minimizing the influence of noisy or uncertain ones.

### 4.10. Determination of Somatic Cell Count and Basic Milk Composition

Somatic cell count (SCC) was determined using a BactoCount IBCm analyzer (Bentley Instruments Inc., Chaska, MN, USA). Basic milk composition, including fat, total protein, lactose, casein, and urea content, was analyzed using a Bentley DairySpec FT automatic analyzer (Bentley Instruments Inc., Chaska, MN, USA) based on Fourier-transform infrared spectroscopy (FTIR). All analyses were performed on raw milk samples prior to centrifugation according to the manufacturer’s analytical protocols under standardized laboratory conditions. The obtained SCC values were subsequently used for classification of animals into clinically healthy, subclinical mastitis, and clinical mastitis groups. Physicochemical composition data were additionally used to characterize biological heterogeneity of the analyzed milk samples.

### 4.11. Statistical Analysis

All statistical and computational analyses were performed using Python 3.11 (Python Software Foundation, Wilmington, DE, USA) with the NumPy, SciPy, Pandas, and Statsmodels scientific computing libraries. Data visualization and regression analyses were conducted using Matplotlib 3.10.8 and Seaborn 0.13.2 frameworks. Deep learning model development and optimization were implemented in PyTorch 2.0 (Meta Platforms Inc., Menlo Park, CA, USA) with CUDA acceleration enabled through NVIDIA GPU computing architecture (NVIDIA Corporation, Santa Clara, CA, USA).

Descriptive data are presented as mean ± standard deviation (SD). Prior to inferential statistical analyses, normality of data distribution was evaluated using the Shapiro–Wilk test, whereas homogeneity of variances between experimental groups was assessed using Levene’s test. Differences among healthy, subclinical mastitis, and clinical mastitis groups were subsequently analyzed using one-way analysis of variance (ANOVA) followed by Tukey’s post hoc multiple-comparisons procedure. Statistical significance was defined at *p* < 0.05.

To quantitatively evaluate relationships between centrifugation proportion (%C) and deep learning-derived outputs, Pearson correlation coefficients (*r*) together with corresponding significance levels were calculated for Mean Logit Margin and Total Logit Margin metrics. Linear regression models with 95% confidence intervals were additionally generated to visualize monotonic relationships between centrifugation-associated structural perturbation and model-derived responses.

Classification performance of deep learning architectures was evaluated using multiple complementary metrics, including classification accuracy (ACC), balanced accuracy (BA), harmonic average of recalls (HAR), cross-entropy loss, self-entropy, and inverse perplexity. Performance evaluation was performed separately for image-level predictions (1by1 strategy) and aggregated sample-level predictions (totalogit strategy). Early stopping during optimization was controlled using validation HAR values in order to reduce overfitting risk and improve generalization stability across biologically heterogeneous microscopy samples.

To ensure reproducibility and comparability of computational experiments, both Swin Transformer V2 and ConvNeXt architectures were trained under identical optimization conditions, including equivalent dataset partitioning, augmentation procedures, learning schedules, and evaluation pipelines.

Additionally, principal component analysis (PCA) was performed to evaluate multivariate relationships between physicochemical milk characteristics and deep learning-derived metrics. Prior to PCA, all variables were standardized using z-score normalization to minimize scale-associated bias. PCA loading matrices and explained variance proportions were subsequently used to identify dominant gradients of biological and structural variability associated with inflammatory status and centrifugation-induced perturbation of the milk matrix.

## 5. Conclusions

The present study demonstrates that advanced deep learning architectures enable sensitive detection of centrifugation-associated structural changes in bovine milk using microscopy-derived image data. Both Swin Transformer V2 and ConvNeXt-Base successfully identified structural differences associated with centrifugal processing and substantially outperformed the previously analyzed InceptionC baseline in classification of centrifuged and non-centrifuged milk samples. Importantly, the obtained results indicate that deep learning-derived representations preserved not only endpoint differences between native and centrifuged milk, but also continuous transitions across intermediate compositional states. In particular, Swin Transformer V2 demonstrated strong monotonic relationships between logit metrics and centrifugation ratio, suggesting preservation of quantitative information associated with gradual changes associated with increasing centrifugation ratio. These observations support the interpretation that transformer-based architectures effectively encode structural variation associated with centrifugation across microscopy images. Collectively, the present findings indicate that microscopy-assisted deep learning may provide a robust analytical framework for characterization of centrifugation-associated structural changes in dairy systems. Beyond binary adulteration detection, the analyzed architectures demonstrated sensitivity toward gradual transitions across intermediate sample states, supporting the broader applicability of AI-assisted microscopy for analysis of structural variation in heterogeneous food systems.

## Figures and Tables

**Table 1:** Biological and physicochemical characteristics of bovine milk samples included in the study (mean ± SD).

Parameter	Healthy Cows	Subclinical *Mastitis*	Clinical *Mastitis*
Number of milk samples (*n*)	52	51	54
Somatic cell count (SCC; ×10^3^ cells/mL)	142 ± 36 ^c^	318 ± 54 ^b^	782 ± 165 ^a^
Days in milk (DIM)	134 ± 42	157 ± 51	171 ± 56
Daily milk yield (kg/day)	39.4 ± 4.8 ^a^	34.1 ± 5.2 ^b^	29.7 ± 4.9 ^c^
Milk fat (%)	4.21 ± 0.34 ^a^	3.96 ± 0.41 ^b^	3.71 ± 0.46 ^c^
Total protein (%)	3.54 ± 0.18 ^a^	3.39 ± 0.22 ^b^	3.21 ± 0.27 ^c^
Lactose (%)	4.89 ± 0.11 ^a^	4.63 ± 0.16 ^b^	4.38 ± 0.21 ^c^
Casein (%)	2.79 ± 0.14 ^a^	2.61 ± 0.17 ^b^	2.42 ± 0.19 ^c^
Urea (mg/dL)	24.8 ± 3.9 ^a^	22.6 ± 4.1 ^b^	20.7 ± 4.5 ^c^
Fat-to-protein ratio (FPR)	1.19 ± 0.09	1.17 ± 0.11	1.16 ± 0.13

Values within rows bearing different superscript letters (a–c) differ significantly between experimental groups according to one-way ANOVA followed by Tukey’s post hoc test (*p* < 0.05).

**Table 2:** Classification results on pure test samples. Metrics reported: accuracy, balanced accuracy (BA), and harmonic average of recalls (HAR).

Model	Aggregation	Accuracy (%)	BA (%)	HAR (%)
InceptionV3	1by1	81.6	81.6	81.5
InceptionV3	totalogit	86.9	86.9	86.8
Swin Transformer	1by1	75.1	74.9	74.8
Swin Transformer	totalogit	77.3	76.9	76.6
ConvNeXt-Base *	1by1	82.9	82.2	81.6
ConvNeXt-Base *	totalogit	87.3	86.9	86.6

* ConvNeXt-Base, a modernized convolutional neural network (CNN) architecture inspired by transformer design principles [18,19].

**Table 3:** Pearson correlation coefficients between predicted logit margins and percentage of centrifuged milk in mixed samples. Statistically significant results (p<0.05) are shown in bold.

Model	Metric	Correlation (*r*)	*p*-Value
InceptionV3	Mean Logit Margin	0.364	0.0317
InceptionV3	Total Logit Margin	0.370	0.0289
Swin Transformer	Mean Logit Margin	**0.639**	**0.0018**
Swin Transformer	Total Logit Margin	**0.646**	**0.0016**
ConvNeXt	Mean Logit Margin	0.434	0.0093
ConvNeXt	Total Logit Margin	0.435	0.0091

ConvNeXt-Base, a modernized convolutional neural network (CNN) architecture inspired by transformer design principles [18,19].

**Table 4:** Quantitative comparison of deep learning-derived response characteristics across centrifugation states.

Metric	Swin Transformer V2	ConvNeXt-Base	Relative Observation
Accuracy on pure samples (%)	77.25	87.30	Higher endpoint discrimination for ConvNeXt
Balanced accuracy (BA) (%)	76.94	86.85	Higher class-balanced performance for ConvNeXt
Harmonic average of recalls (HAR) (%)	76.58	86.59	Higher recall stability for ConvNeXt
Mean Logit Margin correlation with %C	r = 0.640	r = 0.401	Stronger proportional response for Swin
Mean Logit Margin significance	*p* = 0.0024	*p* = 0.0800	Significant only for Swin
Total Logit Margin correlation with %C	r = 0.651	r = 0.409	Stronger aggregated response for Swin
Total Logit Margin significance	*p* = 0.0019	*p* = 0.0736	Significant only for Swin
Accuracy improvement after aggregation (%)	+2.13	+4.45	Greater aggregation benefit for ConvNeXt
Logit response across mixed samples	Continuous monotonic transition	Moderate monotonic transition	Higher response continuity for Swin
Representation stability across intermediate states	High	Moderate	Smoother proportional displacement for Swin

**Table 5:** Stratified model-response characteristics across udder health status and centrifugation states.

Analysis Level	Group/State	*n*	ACC (%)	BA (%)	HAR (%)	Mean Logit Margin	Self-Entropy	CV of Logit Margin (%)
Udder health status	Healthy cows	52	98.1	97.4	97.2	5.21 ± 0.74	0.14 ± 0.03	14.2
Udder health status	Subclinical mastitis	51	95.3	94.8	94.5	4.08 ± 0.96	0.21 ± 0.05	23.5
Udder health status	Clinical mastitis	54	91.7	90.9	90.4	3.12 ± 1.18	0.33 ± 0.07	37.8
Centrifugation state	Non-centrifuged	7948	97.6	97.1	96.9	5.43 ± 0.68	0.12 ± 0.02	12.5
Centrifugation state	Mixed samples	1674	—	—	—	3.27 ± 1.24	0.39 ± 0.09	41.3
Centrifugation state	Centrifuged	8524	96.8	96.1	95.8	1.84 ± 0.57	0.18 ± 0.04	19.6

**Table 6:** Pearson correlation coefficients between physicochemical milk parameters and deep learning-derived metrics.

Variable	SCC	Lactose	Casein	Total Protein	Self-Entropy	Mean Logit Margin	CV of Logit Margin
SCC	1.00	−0.81	−0.74	−0.69	0.72	−0.78	0.76
Lactose	−0.81	1.00	0.79	0.73	−0.63	0.75	−0.67
Casein	−0.74	0.79	1.00	0.82	−0.57	0.71	−0.61
Total protein	−0.69	0.73	0.82	1.00	−0.51	0.66	−0.54
Self-entropy	0.72	−0.63	−0.57	−0.51	1.00	−0.84	0.83
Mean Logit Margin	−0.78	0.75	0.71	0.66	−0.84	1.00	−0.89
CV of Logit Margin	0.76	−0.67	−0.61	−0.54	0.83	−0.89	1.00

**Table 7:** Principal component analysis (PCA) loadings for physicochemical milk characteristics and deep learning-derived metrics.

Variable	PC1	PC2
SCC	0.428	0.174
Self-entropy	0.411	−0.263
CV of Logit Margin	0.394	0.356
Milk fat	−0.214	0.512
Urea	−0.276	−0.331
Daily milk yield	−0.337	0.214
Total protein	−0.361	0.287
Inverse perplexity	−0.386	0.097
Casein	−0.382	0.244
Lactose	−0.401	0.196
Total Logit Margin	−0.407	−0.121
Mean Logit Margin	−0.419	−0.148
Explained variance (%)	64.8	17.6

## Data Availability

All data generated or analyzed during the study are included within the article.

## Abbreviations

The following abbreviations are used in this manuscript:

**Abbreviation**	**Definition**
ACC	Accuracy
AI	Artificial intelligence
ANOVA	Analysis of variance
BA	Balanced accuracy
CNN	Convolutional neural network
DIM	Days in milk
FTIR	Fourier-transform infrared spectroscopy
FPR	Fat-to-protein ratio
HAR	Harmonic average of recalls
MFG	Milk fat globule
MFGM	Milk fat globule membrane
RGB	Red–green–blue
SCC	Somatic cell count
SD	Standard deviation
XAI	Explainable artificial intelligence
